# Morbidity and Mortality After Rib Fracture in Elderly Patients (>65 Years Old) Compared to a Younger Cohort (≤65 Years of Age) at Doctor Hospital Renaissance Health

**DOI:** 10.7759/cureus.30941

**Published:** 2022-10-31

**Authors:** Lord Mvoula, Jeffrey Skubic, David Weaver, Monica Betancourt-Garcia

**Affiliations:** 1 Surgery, Lincoln Medical and Mental Health Center, Bronx, USA; 2 Surgery Institute, Doctor Hospital Renaissance Health, Edinburg, USA; 3 Center of Excellence for Trauma Research in the Border Region, Doctor Hospital Renaissance Health, Edinburg, USA

**Keywords:** in-patient, outcomes, trauma, death, geriatric, rib fractures

## Abstract

Background:Traumatic rib fracture is a major cause of morbidity and mortality. Recent studies highlight the inadequacy of age and the number of rib fractures (NRFs) to assess patients' care needs, which may unnecessarily increase the burden of intensive care unit (ICU) admissions. Therefore, we sought to clarify the clinical outcomes of patients admitted to a level I trauma center with multiple blunt-trauma rib fractures by age and fracture location.

Methods: We performed a retrospective cohort study of patients aged 18-95 admitted to Doctors Hospital at Renaissance Health with multiple rib fractures during 2017-2020. Patients with major vascular/cerebral injuries or emergency surgery from other injuries were excluded. The study population comprised 71 patients aged ≤65 and 53 patients aged >65 years. The primary study outcomes included mortality and non-home discharge. ICU length of stay (ICU-LOS), total hospital length of stay (HLOS), and days on the ventilator were the secondary outcomes. Study outcomes were also analyzed by stratifying patients by fracture location.

Results: Patients aged >65 years with multiple blunt-trauma rib fractures had lower mortality rates despite a higher prevalence of comorbidities but with higher rates of non-home discharges compared to younger patients. However, the mortality and non-home discharge odds ratios were not statistically significant. Also, median ICU-LOS and HLOS were numerically higher in geriatric patients but were not statistically significant. Nonetheless, younger patients required significantly more days of respiratory support than older patients. Similar differences were observed in the clinical outcome of patients ≤65 or >65 years when stratified by fracture locations.

Conclusion: Young patients with blunt trauma rib fractures may have similar, if not worse, clinical outcomes than geriatric patients. These findings underscore the need for individual assessment of the patient's trauma severity independent of age, the number of rib fractures, or fracture location to reduce ICU burden.

## Introduction

About 10% of trauma patients in emergency departments have blunt chest trauma, which remains one of the most common traumatic reasons for hospital presentations globally [1]. Motor vehicle crashes (MVC) and falls are the most common causes of blunt chest trauma [2,3]. The mortality rate in blunt chest trauma patients is estimated at 4-20% [1-4]; however, nearly half of the surviving patients experience prolonged morbidities [5-8]. More recent studies demonstrate that as many as 60% of patients with blunt chest trauma may have traumatic rib fractures [2]. In the United States alone, 22,000 to 45,000 patients are hospitalized with rib fractures annually, costing about $469 million [9].

The management of rib fractures in the geriatric population has received considerable scientific attention due to their higher susceptibility to rib fractures after blunt chest trauma [10-12], with an increased risk of subsequent rib fractures and death [13,14]. However, whether older adults have worse in-hospital or post-discharge outcomes remains controversial. For instance, some studies indicate that younger patients with moderate-to-severe blunt rib trauma are more likely to require post-discharge reevaluation and hospital readmission [15,16], albeit not always consistently [17,18]. In-hospital outcomes of rib fracture patients are also inconsistently reported in the literature. In one study, patients aged ≥65 years had longer intensive care unit length of stay (ICU-LOS) (6.1 vs. 4.0), total hospital length of stay (HLOS) (15.4 vs. 10.7 days), and days on a ventilator (DOV) (4.3 vs. 3.1) [3]. Another study found that when followed long-term, age was not associated with HLOS, ICU-LOS, or quality of life as indicated by the EQ-5D index at three years of follow-up [19]. Additionally, in a large database study, which predominantly included patients aged 51 to 79 years old with traumatic rib fractures, advanced age was associated with lower odds of tracheostomy and shorter HLOS with slightly elevated mortality risk (OR=1.03; 95% CI 1.02-1.03) [20].

Moreover, recent studies indicate that age may not be a good indicator of the risk of in-hospital complications or for identifying patients requiring admission to the ICU. For example, Chien et al. showed that while an injury severity score of 4 or more or a thoracic trauma severity score of 5-7 was associated with greater odds of pulmonary complications, advanced age (≧65) was not (p < 0.80; OR 95 CI 0.45-1.85) [2]. Bankhead-Kendall et al. showed that although patients ≥65 years old were four times more likely to die as a direct result of rib fractures, rib fractures were the immediate cause of death in only 0.5% of the patients admitted to their level I trauma center [21]. Clark et al. attributed higher mortality rates in blunt chest trauma patients to central nervous system injuries, hemorrhage, and concurrent pulmonary contusion with flail chest [22]. Similarly, Jones et al. showed a low mortality rate of 1.8-3.2% in rib fracture patients with no other associated injury with a null association between mortality and the number of rib fractures (NRF) [23]. NRF, which is often used along with advanced age to identify patients requiring intensive care, has low specificity in predicting complications such as pneumothorax, hemothorax, and pulmonary contusion [2] and is not indicative of mortality risk [24]. Instead, the localization of the fracture (i.e., bilateral compared to a one-sided fracture), irrespective of NRF, maybe a better predictor of pulmonary complication rates [2] and the severity of risk fracture [23,24].

Identifying reliable predictors of complication and mortality risks in patients with rib fractures has significant implications for reducing the burden of ICU admission. Therefore, to clarify the impact of advancing age on mortality and in-hospital outcomes, we sought to determine if preexisting comorbidities are associated with ICU-LOS, HLOS, and DOV among blunt-trauma rib-fracture patients admitted to our level I trauma center.

## Materials and methods

Study design, setting, and study participants

Our study was a single-site observational, retrospective cohort study of patients admitted with blunt chest trauma between 2017 and 2020 to the Doctors Hospital at Renaissance Health (DHR Health). DHR Health is a private, single-rehabilitation hospital that became the first and only Level 1 Trauma Center south of San Antonio, Texas. Institutional ethics approval was granted in compliance with the Declaration of Helsinki and the US Federal Policy for the Protection of Human Subjects (Approval number: 1778348-2). Given the study's retrospective nature, we waived the requirement for individual patient consent.

**Figure 1:**
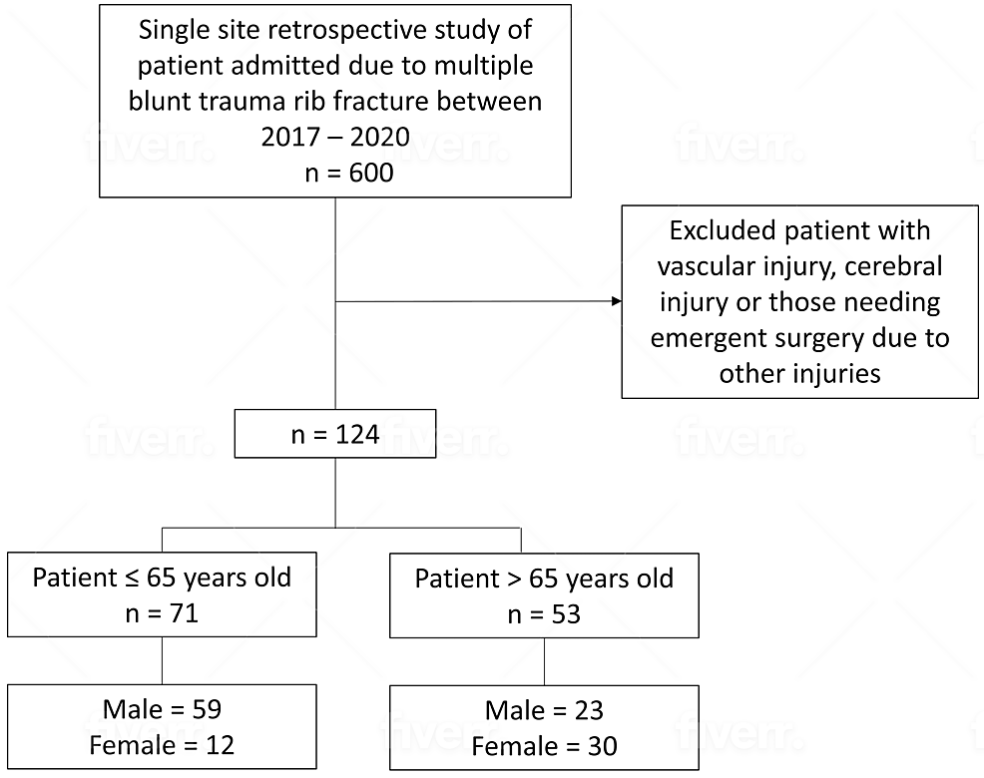
Study design/participant selection.

All adults aged 18 years or older with a diagnosis of multiple rib fractures were initially considered for inclusion (n=600). Patients aged >95 years with a single fractured rib or those treated for fractured ribs in the emergency department without being admitted to the hospital were excluded from our studies. We also excluded patients with vascular injury, cerebral injury, or those needing emergent surgery due to other injuries. The final study population of 124 adults was then dichotomized with 71 patients in the ≤65 years group and 53 patients in the >65 years group (Figure 1). Patients in each group were further divided based on the laterality of the fracture (right, left, and bilaterally). Due to the unavailability of sidedness of the fracture in some of our patients, we added a fourth category (unspecified rib fracture).

Study outcomes

The primary study outcomes included mortality and non-home discharge disposition. The secondary outcomes were ICU-LOS, HLOS, and the number of days on mechanical ventilation.

Statistical analysis

Linear regression was used to determine the odds ratios for mortality and non-home discharges. Statistical significance was tested using Fisher's exact test with a p-value cut-off of <0.05. ICU-LOS, HLOS, and the number of days on mechanical ventilation were presented as percentages, mean ± standard deviation (SD), and median (range), and the difference between the two groups (≤65 years old and >65 years old) was compared using an unpaired Student's t-test or Mann-Whitney test as appropriate. A two-sided p-value of <0.05 was used to indicate statistical significance. The primary and secondary outcomes in geriatric and younger patients were also compared by location of rib fracture (right-sided, left-sided, bilateral, or unspecified rib fractures). All analyses were performed using GraphPad Prism version 9.0.0 for Windows (GraphPad Software, San Diego, California, USA).

## Results

Descriptive analysis

This study includes a total of 124 patients with rib fractures. The mean age of the study population was 58.2±21.4 years. 57.2% were ≤65 years old, and 33.9% were females. The proportion of females was higher in the older (56.6%) than in the younger patients' group (16.9%). Expectedly, geriatric patients had higher prevalence of comorbidities including hypertension (71.7% vs. 25.3%), diabetes mellitus (34.0% vs 15.5%), anticoagulant therapy (15.0% vs. 1.4%), COPD (9.4% vs. 1.4%), and dementia (5.7% vs. 0%); while both groups had similar prevalence of obesity (38.0% vs. 37.7%) and depression (3.8% vs. 4.2%). MVC (motor vehicle collision) was the most common mechanism of injury in younger patients (52.1% vs. 9.4%), while ground-level falls caused most rib fractures in the elderly group (66.0% vs. 11.3%). While the overall mortality rate was higher in <65-year-old patients (5.63%) than in the older cohort (1.89%), we observed higher non-home discharges among older patients (18.4% vs. 24.5%) (Table 1). The median ICU-LOS and HLOS were higher in patients aged ≤65 compared to older patients. Nevertheless, younger patients required more DOV than older patients (Table 1). 45.2% of the study population had right-sided rib fractures. 41.9% had left-sided rib fractures. 8.1% had bilateral, and 4.8% had unspecified rib fractures (Table 1). Table1 stratify patients based on the location of the rib fracture.

**Table 1:** Patient characteristics by location of rib fracture (Fx). *Others include <5% within-group prevalence of ADHD (attention-deficit/hyperactivity disorder), alcoholism, anxiety, bipolar disorder, cirrhosis, chronic renal failure, cardio-vascular attack, end-stage renal disease, epilepsy, functionally dependent health status, hypercholesterolemia, hyperlipidemia, hypothyroidism, mental/personality disorder, obstructive sleep apnea, peripheral arterial disease, post-traumatic stress disorder, and schizophrenia.

	≤65 years old (n=71)				>65 years old (n=53)			
	Right-sided Fx (n=33)	Left-sided Fx (n=30)	Bilateral Fx (n=6)	Unspecified Fx (n=2)	Right-sided Fx (n=23)	Left-sided Fx (n=22)	Bilateral Fx (n=4)	Unspecified Fx (n=4)
Male, %	84.85%	90%	50%	50%	39.13%	45.45%	25%	75%
Female, %	18.18%	10%	100%	50%	65.22%	59.09%	125%	25%
Mean age, years	46.8±10.6	41.3±11.8	28.0±9.8	45.5±18.5	79±8.6	79.9±6.2	79.2±9.3	70.5±4.0
BMI, kg/m^2^	28.8±6.4	32.1±8.7	28.5±6.5	39.6±4.2	27.8±7.4	28.0±6.0	31.9±4.1	30.1±5.3
Mean SBP, mmHg	137.4±22.7	138.2±25.1	120.3±40.3	114.5±22.5	155.0±20.5	145.9±23.3	149.0±25.7	128.2±28.1
Mean DBP, mmHg	82.4±16.8	85.1±24.0	69.0±22.0	69.0±12.0	76.3±15.3	74.9±18.8	79.7±21.1	76.5±18.6
Prevalence of comorbidities (%)								
None	33.33%	26.67%	83.33%	50%	17.39%	4.55%	25%	0%
Obesity	36.36%	50%	0%	0%	30.43%	36.36%	75%	50%
Hypertension	33.33%	20%	0%	50%	65.22%	86.36%	50%	50%
Diabetes militus	18.18%	13.33%	0%	50%	43.48%	27.27%	25%	25%
Anticoagulant therapy	3.03%	0%	0%	0%	21.74%	9.09%	0%	25%
Congestive heart failure	0%	0%	0%	0%	13.04%	4.55%	0%	0%
COPD	3.03%	0%	0%	0%	4.35%	18.18%	0%	0%
Dementia	0%	0%	0%	0%	4.35%	9.09%	0%	0%
Depression	3.03%	6.67%	0%	0%	0%	9.09%	0%	0%
Others*	39.39%	33.33%	16.67%	50%	13.04%	9.09%	0%	0%
Mechanism of injury (%)								
Motor vehicle crash	51.52%	50%	50%	100%	13.04%	4.55%	0%	25%
Same level fall	12.12%	13.33%	0%	0%	65.22%	68.18%	75%	50%
Fall >6'	15.15%	16.67%	0%	0%	13.04%	22.73%	0%	0%
Fall<6'	0%	0%	0%	0%	17.39%	9.09%	25%	25%
Auto ped	6.06%	13.33%	16.67%	0%	0%	4.55%	0%	0%
Bicycle accident	0%	3.33%	0%	0%	0%	4.55%	0%	0%
Firearms	6.06%	0%	16.67%	0%	0%	0%	0%	0%
Blunt trauma	9.09%	0%	0%	0%	0%	0%	0%	0%
Crushing injury	0%	3.33%	16.67%	0%	0%	0%	0%	0%
Mortality, %	0%	3.33%	33.33%	50%	0%	4.55%	0%	0%
Discharge location (%)								
Home	90.91%	90%	50%	0%	91.3%	86.36%	25%	100%
Nursing home	0%	3.33%	0%	0%	8.7%	13.64%	0%	0%
Rehab	3.03%	3.33%	16.67%	0%	8.7%	4.55%	50%	0%
Correctional	0%	0%	0%	50%	0%	0%	0%	0%
Hospice	6.06%	0%	0%	0%	0%	4.55%	25%	0%
AMA	0%	6.67%	0%	0%	0%	0%	0%	0%
Morgue	0%	3.33%	33.33%	50%	0%	4.55%	0%	0%

Primary and secondary outcomes

Regarding the primary study outcomes, while the overall mortality rate was higher in <65-year-old patients (5.63%) than in older patients (1.89%), we observed higher non-home discharges among older patients (18.4% vs. 24.5%) (Table 2). However, the odds ratios for mortality and non-home discharges were not statistically significant. Furthermore, although the median days of ICU and total hospital LOS range were wider and the means numerically higher in patients aged ≤65 compared to older patients, the differences were not statistically significant (Table 2). Nevertheless, younger patients required significantly more days of ventilator use than older patients (p=0.0103).

**Table 2:** Univariate analysis of primary and secondary outcomes. The p-value for odds ratios was compared using Fisher's exact test, mean±SD was compared using Student's t-test, and the differences in medians were compared using Mann Whitney test. *Denotes statistical significance. ICU-LOS: intensive care unit length of stay; HLOS: total hospital length of stay.

	≤65 (n=71)	>65 (n=53)	OR (95%CI)	p-value
Primary outcomes				
Mortality, %	5.63%	1.89%	3.1 (0.49–38.63)	0.39
Non-home discharge, %	18.4%	24.5%	1.4 (0.60–3.31)	0.50
Secondary outcomes				
ICU-LOS [mean±SD], days	1.9±4.6	0.8±2.5	-	0.11
ICU-LOS [median (range)], days	0 (0–24)	0 (0–14)	-	0.08
HLOS [mean±SD], days	6.4±7.2	5.7±5.6	-	0.58
HLOS [median (range)], days	5 (0–48)	4 (1–32)	-	0.98
Ventilator use [mean±SD], days	1.7±5.3	0.3±1.5	-	0.08
Ventilator use [median (range)], days	0 (0–35)	0 (0–9)	-	0.0103*

Similar findings (primary and secondary outcomes) were noted when patients were grouped by fracture location (Table 3). The odds ratios for mortality and non-home discharges were statistically insignificant for all fracture locations. ICU LOS, total LOS, and days on the ventilator were also statistically insignificant for all fracture locations (Table 3), with two clinically inconsequential exceptions. The mean ICU LOS (p=0.008) and mean days on the ventilator (p=0.0012) were significantly longer in younger patients with unspecified rib fractures than in older patients with unspecified fracture locations. However, the median days for ICU LOS or ventilator use were not statistically significant between the younger and older groups of patients with unspecified rib fracture locations (Table 3).

**Table 3:** Univariate analysis of primary and secondary outcomes by fracture (Fx) location. The p-value for odds ratios was compared using Fisher's exact test, mean±SD was compared using Student's t-test, and the differences in medians were compared using Mann Whitney test. *Denotes statistical significance. ICU-LOS: intensive care unit length of stay; HLOS: total hospital length of stay.

	Right sided Fx				Left-sided Fx				Bilateral Fx				Unspecified Fx			
Primary outcomes	OR (95%CI)		p		OR (95%CI)		p		OR (95%CI)		p		OR (95%CI)		p	
Mortality	0.72 (∞)		>0.99		0.72 (0.04–14.36)		>0.99		∞ (0.32-∞)		0.47		∞ (0.22-∞)		0.33	
Non-home discharge	2.1 (0.51-8.96)		0.43		2.44 (0.67–8.45)		0.29		3.00 (0.23-50.28)		0.57		0 (0.00–0.73)		0.07	
Secondary outcomes	≤65 (n=33)	>65 (n=23)		p	≤65 (n=30)	>65 (n=22)		p	≤65 (n=6)	>65 (n=4)		p	≤65 (n=2)	>65 (n=4)		p
ICU-LOS [mean±SD], days	2.2±5.5	0.7±3		0.24	1.1±3.3	0.9±2.4		0.75	3.2±5.2	1±2		0.46	3±1.4	0±0		0.008*
ICU-LOS [median (range)], days	0 (0-24)	0 (0–14)		0.15	0 (0–16)	0 (0–11)		0.93	0.5 (0-13)	0 (0–4)		0.37	3 (2–4)	0 (0–0)		0.0667
HLOS [mean±SD], days	6.2±5.6	5.2±5.1		0.48	6.4±8.9	6.2±7		0.94	8.2±8.0	7.8±1.7		0.92	4.5±3.5	4.5±3.3		>0.9999
HLOS [median (range)], days	6 (0–25)	3 (1–25)		0.52	4 (1–48)	3.5 (1–32)		0.96	6 (1–19)	7.5 (6–10)		0.94	4.5 (2–7)	3.5 (2–9)		>0.9999
Ventilator use [mean±SD], days	1.8±4.9	0.3±1.3		0.16	1.3±6.4	0.5±1.9		0.56	2.5±2.9	0±0		0.13	2.5±0.7	0±0		0.0012*
Ventilator use [median (range)], days	0 (0–23)	0 (0–6)		0.16	0 (0–35)	0 (0–9)		0.72	1.5 (0–7)	0 (0–0)		0.10	2.5 (2–3)	0 (0–0)		0.07

Patients discharged to the morgue had a mean injury severity score (ISS) of 21.33 ± 16.50 in the younger group (<65 years of age) compared to 30 ± 1.23 in those >65 years of age. Among the patients who did not expire, the mean ± SD injury severity score was 13.17 ± 7.66 in those younger than 65 compared to 8.47 ± 4.08 in the elderly group (Table 4). As noted below, the mean ISS in our mortality group was significantly higher than in the non-mortality group (p < 0.05)

**Table 4:** Univariate analysis of primary outcomes (mortality) based on the injury severity scale. The p-value for odds ratios was compared using Fisher's exact test, Mean ± SD was compared using Student's t-test, and the differences in medians were compared using Mann Whitney test. *Denotes statistical significance.

	Mean ± SD injury severity score in the non-mortality group (n = 130)	Injury severity score in patients with mortality (n = 4)	P value
Patients <65 years of age	13.17 ± 7.66	21.33 ± 16.50	0.1308*
Patients >65 years of age	8.47 ± 4.08	30 ± 1.23	<0.001*

## Discussion

Patients ≤65 years old had a higher mortality rate (5.63%) compared to geriatric patients (1.89%) despite a higher prevalence of comorbidities in the latter. This may be because most patients with mortality had, on average, a higher injury severity score than their counterparts (Table 4). However, it is important to note that there were patients in the non-mortality group with worse injury severity scores. Despite having lower mortality, older patients had higher rates of non-home discharges than younger patients. Nonetheless, our study's mortality and non-home discharge odds ratios were not statistically significant. These findings are inconsistent with mortality rates reported from other level I trauma centers in the United States. For instance, Bulger et al. [3] reported a 22% mortality in the elderly versus 10% in younger patients with rib fractures, and Stawicki et al. [25] reported mortality rates of 20.1% and 11.4%, respectively. Similarly, Pape et al. reported mortality rates of 10.2%, 4.9%, 17.3%, and 30.9% with one rib fracture, 2-3 rib fractures, >3 unilateral rib fractures, and >3 bilateral rib fractures in a population mainly comprising young adults with chest trauma admitted to the Department of Trauma Surgery at Hannover Medical School between 1975 and 1999 [26]. Mortality rates were even higher among patients with contaminant hemopneumothorax [26].

Bulger et al. [3] and Stawicki et al. [25] also reported much longer HLOS, ICU-LOS, and days of ventilator use in young and elderly patients than observed at our level I trauma center, with a significantly longer duration in the older cohort. In our study, the median ICU-LOS and HLOS were higher in geriatric patients but not statistically significant. Nonetheless, younger patients required significantly more days of respiratory support than older patients. This finding could be due to the current practice of intubating intoxicated patients with a GSC < 10 and an appropriate mechanism of injury (i.e., motor vehicle collision). Despite having a similar ISS, most patients <65 years of age were admitted after being involved in a motor vehicle collision compared to the admission diagnosis of ground-level fall in the older cohort (Table 1).

It is worth noting that the mortality rates and LOS durations reported by Bulger et al. [3], Stawicki et al. [25], and Pape et al. [26] are from two decades ago. Novel ways of managing rib fractures, such as multimodal pain control, surgical stabilization of rib fractures, and improved triage pathways, have emerged since then, which may explain, at least in part, the lower mortality rates and shorter LOS durations observed in our study [27]. The lower mortality rates and LOS durations observed at our study site could also be because we excluded patients with significant vascular or cerebral injuries and those needing emergent surgery due to other injuries. This exclusion was essential to assess the outcome in patients with isolated rib fractures. Indeed, studies have shown that cerebral, cardiac, and hepatic injuries are critical determinants of 24-hour mortality after thoracic trauma [26,28]. Accordingly, recent nationwide studies by Peek et al. [29] and Kishawi et al. [30] reported mortality rates of 2.1-2.5% among patients with isolated rib fractures with HLOS, LOS, and DOV similar to those observed in our study [29]. Furthermore, newer studies are indicating that NRF is not associated with worst mortality, HLOS, ICU-LOS, or days on mechanical ventilation [31-32], unlike those of Bulger et al. [3], Stawicki et al. [25], and Pape et al. [26]. Their studies all noted a positive association between NRF and mortality.

Moreover, some studies show that the threshold for NRF for patient prognosis differs by outcome [33,34]. A recent analysis of the National Trauma Data Bank by Shulzhenko et al. [34] identified NRF thresholds of ≥8 fractures to predict increased mortality risks, the need for mechanical ventilation, and a longer duration of mechanical ventilation in geriatric patients with blunt chest trauma. In comparison, the threshold for ICU stay and longer HLOS was ≥5 fractures [34]. The risk of pneumonia complications only increased past seven rib fractures [34].

There is also growing recognition that rib fracture location and functional status may assist in better assessing a patient's care needs while still in the trauma bay. For instance, Haines et al. [35] showed that lateral rib fracture predicts higher mortality risks after controlling for age, gender, and concurrent injuries. In contrast, patients with posterior rib fractures had a higher mortality risk only if they were ≥45 years of age or had ≥3 broken ribs [35]. Relatedly, thoracic trauma patients with an admission systolic blood pressure of under 90 or with bilateral rib fractures may have a greater need for ICU care [36]. However, our study did not observe any clinically relevant difference in mortality, discharge deposition, HLOS, ICU-LOS, or days on ventilator between young and geriatric patients when stratified by rib fracture location (right-sided, left-sided, bilateral, or unspecified rib fractures).

The current study's findings and observations of prior studies discussed above underscore the need for individual assessment of a patient's trauma severity independent of age, NRF, or rib fracture location. In order to reduce the ICU admission burden, researchers have identified several markers beyond age and NRF to identify patients with higher complication risks. For instance, Chrysou et al. [37] showed that injury severity measured using the Thoracic Abbreviated Injury Scale (AIS) was predictive of ICU admissions even though AIS was not associated with ICU-LOS, intubation days, rates of complications, or mortality. Brandon et al. [38] reported that RibScore, a radiographic score based on fracture pattern which includes six variables (namely ≥ 6 rib fractures, bilateral fractures, flail chest, ≥ 3 severely displaced fractures, first rib fracture, and at least one fracture in anterior, lateral, and posterior areas), was associated with complications and adverse pulmonary outcomes. More recently, Billings et al. [39] and Khan et al. [40] showed that rib fracture patients triaged based on forced vital capacity had shorter ICU-LOS and HLOS with 78% lower odds of ICU transfer.

Revised triage guidelines at the Virginia Commonwealth University Hospital, Richmond, VA, USA, emphasized the patient's injury severity and de-emphasize the patient's age and NRF, thereby reducing ICU admission from 73.4% to 62.5% [41]. The revised guidelines also reduced the 48-hour discharge rate by 40%, indicating that ICU resources were available to patients who needed them the most [41]. Importantly, only initial incentive spirometer effort of less than 750 mL and dyspnea were associated with the adverse composite outcome comprising unplanned ICU transfer, difficult pain control, respiratory complication, or death [41]. Advanced age, three or more fractured ribs, unstable chest wall, hemothorax, pneumothorax, pulmonary contusions, concomitant abdominal injuries, and baseline comorbidities were not associated with the adverse composite outcome [41]. Similarly, Butts et al. [27] showed that incentive spirometry is a better predictor of pulmonary complications in patients with rib fractures than NRF or the presence of pulmonary comorbidities. A finding consistent with earlier studies which suggested that decreased spirometric volume is highly predictive of a severe torso injury [42]. Consequently, there is a growing consensus for the use of incentive spirometry to guide the prognosis and care of rib fracture patients [43].

Moreover, there is direct evidence suggesting that advanced age alone does not warrant ICU admission of patients with multiple rib fractures. Naar et al. [44] showed that most geriatric patients with multiple blunt-trauma rib fractures could be safely managed on the floor. In conclusion, in-patient outcomes, including LOS, tracheostomy, and mortality, are significantly worse among older patients with iatrogenic rib fractures than blunt trauma rib fractures [45], suggesting that older patients' functional status may play a more central role in determining the outcomes of blunt trauma rib fractures.

Lastly, we considered the financial implications of ICU admission. Studies done during COVID established that the cost of an ICU stay is around $2902 [46] and that older age, mechanical ventilation, and comorbidities were the primary drivers of cost and hospital length of stay. Unlike most patients in our older cohort group who had Medicare, patients in the younger cohort had a greater financial burden.

There are several limitations to our study. First, given the study's retrospective nature, our findings are hypothesis-generating and need to be confirmed in prospective clinical trials. Second, it is plausible that the observation of this study is influenced by increased attention toward geriatric patients admitted to the study site. Third, we did not assess gender differences between groups. Even though men have poorer clinical outcomes resulting from rib fractures than women [19,47], rib fracture is a significant predictor of future fractures of the rib, wrist, and spine in women, irrespective of age [48]. Lastly, given that this is a single-centered study with a small sample size, there is an inherent risk of Type II error for mortality. Therefore, our findings must be validated in multi-centered studies with a larger sample size.

## Conclusions

In conclusion, this study expands the growing body of evidence that advanced age, NRF, or rib fracture location cannot be used as the sole criteria to assess the risk of in-hospital or post-discharge complications in patients with multiple blunt-trauma rib fractures, which may inadvertently disadvantage younger patients needing intensive care. In our study, elderly patients with blunt trauma rib fractures admitted at Doctor Hospital Renaissance between 2017 and 2020 had better outcomes (non-significant) than patients younger than 65 admitted during the same period. Therefore, efforts to decrease rib morbidity, mortality, and, by proxy, cost should focus on other factors, such as a patient's functional status, injury mechanisms, trauma severity, and comorbidities.
